# Traditional Chinese medicine as an adjunctive therapy improves cardiac function and reduces serum inflammatory markers in patients with chronic heart failure

**DOI:** 10.5937/jomb0-54440

**Published:** 2025-06-13

**Authors:** Yonghong Zheng, Chunhui Huang, Wei Zhang, Yiping Shi, Fangchao Chen, Jianru Zhou

**Affiliations:** 1 Liyang Hospital of Traditional Chinese Medicine, Department of Cardiology, Liyang City, Jiangsu Province, China

**Keywords:** chronic heart failure, traditional Chinese medicine, cardiac function, hronična srčana insuficijencija, tradicionalna kineska medicina, srčana funkcija

## Abstract

**Background:**

To evaluate the impact of traditional Chinese medicine treatment versus standard treatment on cardiac function metrics, serum inflammatory markers, and quality of life in patients with chronic heart failure (CHF).

**Methods:**

A total of 40 CHF patients were randomly assigned to either the observation group (TCM treatment) or the control group (standard Western therapy), with 20 patients in each group. Over a 3-month treatment period, primary outcomes including cardiac function indicators (ejection fraction [EF], cardiac output [CO], left ventricular end-diastolic pressure [LVEDP]), exercise tolerance (6-minute walk test [6MWT] results), and inflammatory markers (high-sensitivity C-reactive protein [hs-CRP], interleukin-6 [IL-6], tumor necrosis factor-alpha [TNF-a]) were assessed. Secondary outcomes included the Minnesota Living with Heart Failure Questionnaire (MLHFQ) scores and adverse reaction rates. Statistical analysis was performed using t-tests and chi-square tests, with significance set at P < 0.05.

**Results:**

After treatment, the observation group showed significantly greater improvements in EF (44.14% ± 4.95% vs. 40.15% ± 4.77%, P = 0.013), CO (4.62 ± 0.76 L/min vs. 4.10 ± 0.72 L/min, P = 0.032), LVEDP (18.76 ± 2.50 mmHg vs. 20.80 ± 2.64 mmHg, P = 0.016), and 6MWT results (526.84 ± 49.20 m vs. 432.75 ± 37.26 m, P < 0.001), compared to the control group. Inflammatory markers and MLHFQ scores were also significantly improved, while the adverse reaction rate was lower in the observation group (0.00% vs. 20.00%, P = 0.035).

**Conclusions:**

TCM as an adjunctive therapy demonstrates superior efficacy and safety compared to standard treatment for CHF, with significant improvements in cardiac function, exercise tolerance, and inflammatory markers. These findings provide quantitative evidence supporting the clinical application of TCM in CHF management.

## Abstract (sr)

**Uvod:**

Cilj rada je bio da se proceni uticaj tradicionalnog lečenja kineske medicine u odnosu na standardni tretman na metriku srčane funkcije, serumske inflamatorne markere i kvalitet života kod pacijenata sa hroničnom srčanom insuficijencijom (CHF).

**Metode:**

Ukupno 40 pacijenata sa CHF nasumično je raspoređeno u grupu za posmatranje (TKM tretman) ili kontrolnu grupu (standardna Vestern terapija), sa 20 pacijenata u svakoj grupi. Tokom perioda lečenja od 3 meseca, primarni ishodi uključujući indikatore srčane funkcije (ejekciona frakcija [EF], minutni volumen [CO], krajnji dijastolni pritisak leve komore [LVEDP]), tolerancija na vežbanje (6-minutni rezultati testa hoda [6MVT]) i inflamatorni markeri (visoka osetljivost, interaktivni C-ukin-6-RP protein, C-ukin-6) faktor nekroze tumora-alfa [TNF-a]). Sekundarni ishodi uključivali su Upitnik za život sa srčanom insuficijencijom u Minesoti (MLHFK) i stope neželjenih reakcija. Statistička analiza je izvršena korišćenjem t-testova i hi-kvadrat testova, sa značajnošću postavljenom na P < 0,05.

**Rezultati:**

Nakon tretmana, posmatračka grupa je pokazala značajno veća poboljšanja u EF (44,14% ± 4,95% na -spram 40,15% ± 4,77%, P = 0,013), CO (4,62 ± 0,76 L/min naspram 4,10 ± 0,72 L/min, P = 70,70 L/min, P = 80). 2,50 mmHg naspram 20,80 ± 2,64 mmHg, P = 0,016) i rezultati 6MVT (526,84 ± 49,20 m vs. 432,75 ± 37,26 m, P < 0,001), u poređenju sa kontrolnom grupom. Inflamatorni markeri i MLHFK rezultati su takođe značajno poboljšani, dok je stopa neželjenih reakcija bila niža u posmatranoj grupi (0,00% naspram 20,00%, P = 0,035).

**Zaključak:**

TCM kao dodatna terapija pokazuje superiornu efikasnost i sigurnost u poređenju sa standardnim tretmanom za CHF, sa značajnim poboljšanjima u srčanoj funkciji, toleranciji na vežbanje i inflamatornim markerima. Ovi nalazi pružaju kvantitativne dokaze koji podržavaju kliničku primenu TCM u lečenju CHF.

## Introduction

Chronic heart failure (CHF) forms the pinnacle of myriad cardiac diseases. With the current statistics denoting prevalences of heart failure ranging from 1.26%–6.70% in the United States; this paints a stark picture of the magnitude of this cardiac condition [1]. Looking globally, China chronicles an even more accentuated presence of the disease- with over 4.5 million diagnoses, thus underscoring its ubiquity. Due to a confluence of unhealthy lifestyle choices and an aging population, CHF manifests as a threat in contemporary medical landscapes [1].

Standard treatments, including angiotensin receptor neprilysin inhibitors (ARNIs), beta-blockers, and diuretics, have improved clinical outcomes but remain limited by significant adverse effects and only partial symptom relief. These limitations highlight the urgent need for complementary or alternative therapies to optimize patient care [2] [3] [4]. One such promising modality is traditional Chinese medicine (TCM), which has been crucial in the treatment of CHF. Traditional Chinese medicine, with its holistic regulatory effects and centuries-long clinical application, has shown promise in CHF management. TCM is believed to target the pathophysiological mechanisms underlying CHF, including inflammation, myocardial remodeling, and impaired cardiac function. For instance, studies have demonstrated that Astragalus-based formulas improve cardiac function and reduce inflammatory marker levels, while bitter ginseng enhances myocardial oxygenation and inhibits inflammation [2] [5]. However, most existing research lacks rigorous comparative evaluation of TCM’s efficacy relative to standard treatment, and the mechanisms underlying its therapeutic effects remain underexplored.

Despite the mounting popularity and success of traditional Chinese medicine in the management of CHF [6] [7], it becomes apparent that adequate comparative research is lacking. There exists a dearth of rigorous scientific scrutiny that contrasts traditional Chinese medicine’s efficacy and superiority compared to standard Western medicine. This research gap poses a critical challenge that this study hopes to address. This study aimed to compare the effects of TCM and standard therapy on cardiac function, inflammatory markers, and quality of life in CHF patients. The overarching goal is to elucidate the scientific community on effective treatment regimens and furnish a substantial basis for clinical decision-making regarding optimal treatment for CHF, thus contributing to the overall improvement in health outcomes for the affected patient population.

## Materials and methods

### Study subjects

The sample for this study was composed of 40 patients diagnosed with Congestive Heart Failure (CHF) who were treated in our cardiovascular department from January 2021 to August 2023. A total of 40 patients diagnosed with chronic heart failure (CHF) were enrolled and randomly assigned to either the observation group (TCM treatment) or the control group (standard treatment), with 20 patients in each group. Randomization was performed using a computer-generated random number table, ensuring allocation concealment by employing sealed, opaque envelopes.

Inclusion criteria include: (1) CHF diagnosis confirmed through clinical symptoms and echocardiogram; (2) presence of dyspnea of varying degrees; (3) classification between II-IV according to the New York Heart Association (NYHA) functional grading; (4) fulfillment of the traditional Chinese medicine diagnosis of Qi-deficiency and blood stasis in chronic heart failure, with primary symptoms like dull complexion, flank pain, fatigue, reluctance to talk, cold extremities and cyanosis of digits, and secondary symptoms like epigastric fullness, hepatospleno megaly, somnolence, pale tongue with purple spots, and a deep and rough pulse; (5) informed consent signed by the patient or their family members; (6) approval from the hospital’s ethics committee.

Exclusion criteria consisted of: (1) co-existing severe arrhythmias, hepatic or renal failure, malignant tumors, or other systemic diseases; (2) CHF as a result of extracardiac factors such as anemia-induced cardiomyopathy or thyrotoxic heart disease; (3) noncompliance or withdrawal from the study or incomplete information; (4) contraindications to the drugs under investigation; (5) breastfeeding or pregnant women.

### Methods

### Observation group

The observation group was treated with traditional Chinese medicine, and was given Liver-sparing and Blood-activating Formula, which was obtained from the pharmacy of Liyang Hospital of Traditional Chinese Medicine, and consisted of 15 g of Chaihu, 10 g of Xiangfu, 30 g of Astragalus, 15 g of Cornu Cervi Pantotrichum, 10 g of Maidong, 10 g of seaweed, 10 g of Pu huang, 30 g of Lutong, 10g of peach kernel, 10 g of safflower, 10 g of Rhizoma Ligustici Chuanxiong, and 10 g of Bitter ginseng, and the above medicines were added to an appropriate amount of water to make the juice of the medicines of 400 mL, which was divided into 2 times of morning and evening oral administration, and was administered as 1 dose per day, and the duration of the therapy was 3 months.

### Control Group

The control group received standard Western medical treatment, consisting of 100 mg bid of Sacubitril/Valsartan, 20 mg qd of Spironolactone, 20 mg qd of Furosemide, and 23.75 mg qd of Metoprolol Succinate CR Tablet for a period of three months.

### Observation indices

Baseline data: Including gender, age, illness duration, functional classification of heart failure and primary disease type. Clinical efficacy: The efficacy was classified as effective, improving by two functional classes or more; somewhat effective, improving by one functional class; and ineffective, no improvement or worsening. Total effective rate = (number of effective cases + number of somewhat effective cases) / total cases x 100%. Minnesota Living with Heart Failure Questionnaire (MLHFQ) score: Evaluated before and after treatment, answering 21 questions about emotional well-being and physical condition. Each item was scored on a scale from 0 (none) to 5 (very much), with lower scores denoting better quality of life. 6-minute walk test (6MWT): Patients were encouraged to walk as far as possible in 6 minutes both before and after treatment, slowing or resting as needed and halting exercise if crucial symptoms emerged. Cardiac Function: Echocardiography was performed before and after the treatment, noting the cardiac output (CO), left ventricular ejection fraction (LVEF), ejection fraction (EF) and left ventricular end-diastolic pressure (LVEDP). Serum Inflammatory Biomarkers: Peri pheral venous blood was collected before and after treatment; hypersensitive C-reactive protein (hsCRP) was tested by immunoturbidimetry, and interleukin-6 (IL-6) as well as tumor necrosis factor-alpha (TNF-α) were measured by enzyme-linked immuno sorbent assay (ELISA). The levels of N-terminal probrain natriuretic peptide (NT-proBNP): Before and after treatment, 5 mL of fasting venous blood was extracted, centrifuged to obtain serum, and then the levels of NT-proBNP were measured using electrochemiluminescence immunoassay. Adverse Effects: Including dizziness, headache, nausea, vomiting, abdominal pain, and diarrhea. The proportion of patients experiencing each type of adverse effect was calculated for both groups.

### Statistical analysis

Data were analyzed using Statistic Package for Social Science (SPSS) 25.0 statistical software (IBM, Armonk, NY, USA). Categorical data were presented as [n(%)]. When the sample size was more than or equal to 40, but the theoretical frequency was 1 T<5, Chi-square test with Yates’ correction was employed. When the sample size was less than 40 or when theoretical frequency T<1, Fisher’s exact probability method was applied for statistical analysis. For metric data conforming to a normal distribution, it was expressed as (±s). For data not conforming to a normal distribution, variable transformations were utilized to achieve normal distribution for statistical analysis using the t-test. A *P*-value of less than 0.05 was considered statistically significant.

## Results

### Comparison of baseline data between two groups

As shown in Table 1, the observation group consists of 12 males and 8 females, aged between 50 to 72 years, with an average age of 62.35±5.68 years. The NYHA classification: 8 cases in class II, 7 cases in class III, and 5 cases in class IV. The underlying diseases are: 10 cases of coronary heart disease, 7 cases of hypertensive heart disease, 2 cases of dilated cardiomyopathy, and 1 case of rheumatic heart disease. The control group includes 13 males and 7 females, aged between 50 to 70 years, with an average age of 62.50±5.44 years. The NYHA classification: 7 cases in class II, 7 cases in class III, and 6 cases in class IV. The underlying diseases are: 11 cases of coronary heart disease, 5 cases of hypertensive heart disease, 2 cases of dilated cardiomyopathy, and 2 cases of rheumatic heart disease. There was no statistically significant difference between the two groups in terms of gender, NYHA classification, underlying diseases, and other baseline data (*P* > 0.05), suggesting that the groups are comparable.

**Table 1:** Comparison of Baseline Data between the Two Groups.

Groups	Gender [n(%)]		Age<br>(years)	Disease<br>Duration<br>(years)	NYHA Classification [n(%)]			Original Disease [n(%)]			
	Male	Female			Stage <br>II	Stage<br>III	Stage<br>IV	Coronary<br>Heart<br>Disease	Hypertensive<br>Heart <br>Disease	Dilated<br>Cardiomyopathy	Rheumatic<br>Heart <br>Disease
Observation<br>Group<br>(n=20)	12(60.00)	8(40.00)	62.35±5.68	1.65±0.38	8(40.00)	7(35.00)	5(25.00)	10(50.00)	7(35.00)	2(10.00)	1(5.00)
Control Group<br>(n=20)	13(65.00)	7(35.00)	62.50±5.44	1.70±0.35	7(35.00)	7(35.00)	6(30.00)	11(55.00)	5(25.00)	2(10.00)	2(10.00)
* χ^2^/t *	0.107		0.087	0.433	0.158			0.714			
* P *	0.744		0.931	0.668	0.924			0.870			

### Comparison of clinical efficacy between two groups

As depicted in Table 2, the observation group demonstrated significant improvement in 11 cases, progress in 8 cases, and no effect in 1 case, producing an overall effectiveness rate of 95.00%. The control group showed substantial improvement in 7 cases, improvement in 7 cases, and no effect in 6 cases, yielding an overall effectiveness rate of 60.00%. A statistically significant difference was noted when comparing the overall effectiveness rates of the two groups (*P*<0.05), with the observation group surpassing the control group. This suggests that, compared to standard treatment, TCM treatment can enhance the therapeutic effects in patients with CHF.

**Table 2:** Comparison of Clinical Efficiency Between Two Groups [n(%)].

Group	Remarkable Effect	Improvement	No Effect	Overall Efficacy Rate
Observation Group (n=20)	11 (55.00)	8 (40.00)	1 (5.00)	19 (95.00)
Control Group (n=20)	7 (35.00)	7 (35.00)	6 (30.00)	14 (60.00)
χ2				4.329
P				0.037

### Comparison of MLHFQ scores before and after treatment between two groups

As represented in Table 3, there was no statistically significant difference in the MLHFQ (Minnesota Living with Heart Failure Questionnaire) scores between control and observation groups prior to treatment (*P*>0.05). However, a significant difference was observed in MLHFQ scores post-treatment (*P*<0.05), wherein the observation group recorded lower scores compared to the control group. This suggests that, when compared to the standard treatment, TCM treatment may further enhance the quality of life for patients with CHF.

**Table 3:** Comparison of Pre and Post-treatment MLHFQ Scores Between Two Groups (*x̅*±s, score).

Group	Pre-treatment	Post-treatment
Observation<br>Group (n=20)	57.18±9.25	36.32±4.28
Control Group<br>(n=20)	57.62±9.14	43.90±6.25
t	0.151	4.475
P	0.881	0.001

### Comparison of 6MWT results before and after treatment between two groups

As illustrated in Table 4, there was no statistically significant difference in the results of the 6-Minute Walk Test (6MWT) between control and observation groups prior to treatment (P>0.05). However, posttreatment comparison of the 6MWT revealed a statistically significant difference (P<0.05). Notably, the observation group displayed a longer 6MWT result compared to the control group after treatment. This suggests that, when compared to standard treatment, TCM treatment can potentially enhance the exercise tolerance of patients with CHF.

**Table 4:** Comparison of Pre and Post-Treatment 6MWT Results Between Two Groups (*x̅*±s, m).

Group	Pre-treatment	Post-treatment
Observation<br>Group (n=20)	349.53±25.74	526.84±49.20
Control Group<br>(n=20)	350.82±26.98	432.75±37.26
t	0.155	6.818
P	0.878	0.001

### Comparison of cardiac function indicators before and after treatment between two groups

As shown in Table 5, there were no statistically significant differences in cardiac function indicators such as CO, EF, and LVEDP between control and observation groups before treatment (P > 0.05). However, after treatment, there were statistically significant differences in CO, EF, and LVEDP between the two groups (P < 0.05). Specifically, the observation group exhibited higher CO and EF and lower LVEDP compared to the control group after treatment, indicating that TCM treatment may better improve the cardiac function of patients with CHF compared to standard treatment.

**Table 5:** Comparison of Pre- and Post-Treatment Cardiac Function Parameters Between Two Groups (*x̅*±s).

Group	CO (L/min)		EF (%)		LVEDP (mmHg)	
	Pre-treatment	Post-treatment	Pre-treatment	Post-treatment	Pre-treatment	Post-treatment
Observation Group (n=20)	3.85±0.40	4.62±0.76	36.22±4.26	44.14±4.95	25.45±3.24	18.76±2.50
Control Group (n=20)	3.90±0.38	4.10±0.72	36.45±4.38	40.15±4.77	25.28±3.16	20.80±2.64
* t *	0.405	2.221	0.168	2.596	0.168	2.509
* P *	0.688	0.032	0.867	0.013	0.867	0.016

### Comparison of serum inflammatory markers and NT-BNP levels before and after treatment between two groups

As presented in Table 6, there was no significant statistical difference in the serum levels of inflammation markers such as high-sensitivity CReactive Protein (hs-CRP), Interleukin-6 (IL-6), Tumor Necrosis Factor-alpha (TNF-α) and NT-BNP between the two groups prior to treatment (P>0.05). However, post-treatment comparison of these serum inflammation markers revealed a significant statistical difference (P<0.05). Specifically, the observation group showed lower serum levels of hs-CRP, IL-6, TNF-α and NT-BNP compared to the control group, suggesting that compared to standard treatment, TCM treatment may more effectively reduce serum inflammation marker levels in patients with CHF.

**Table 6:** Comparison of Pre and Post-Treatment Serum inflammatory markers and NT-BNP Levels Between Two Groups (*x̅*±s, ng/L).

Group	hs-CRP (ng/L)		IL-6 (ng/L)		TNF-α (ng/L)		NT-BNP (pg/mL)	
	Pre-treatment	Post-treatment	Pre-treatment	Post-treatment	Pre-treatment	Post-treatment	Pre-treatment	Post-treatment
Observation<br>Group<br>(n=20)	12.85±2.74	5.86±1.25	86.52±10.25	35.62±5.72	37.50±5.04	14.65±3.20	2015.40±210.38	856.54±150.32
Control<br>Group <br>(n=20)	12.92±2.80	9.14±1.52	85.10±10.74	52.88±5.90	37.62±5.18	24.32±3.95	2014.95±212.45	960.34±150.44
t	0.080	7.454	0.428	9.393	0.074	8.507	0.007	2.183
P	0.937	0.001	0.671	0.001	0.941	0.001	0.995	0.035

### Comparison of adverse reaction incidence between two groups

As per Table 7, there were no cases of adverse reactions in the observation group, resulting in an incidence rate of 0.00%. The control group recorded one case each of symptoms like dizziness, headache, nausea, vomiting, abdominal pain, and diarrhea, which calculated to an incidence rate of 20.00%. There was a significant statistical difference in the incidence of adverse reactions between the two groups (*P*<0.05). Specifically, the incidence of adverse reactions was lower in the observation group compared to the control group. This finding suggests that, compared to standard treatment, TCM treatment reduces the incidence of adverse reactions and enhances the safety for patients with CHF.

**Table 7:** Comparison of Adverse Reaction Incidence Between Two Groups [n(%)].

Group	Dizziness	Headache	Nausea and<br>Vomiting	Abdominal Pain<br>and Diarrhea	Incidence<br>Rate
Observation Group (n=20)	0 (0.00)	0 (0.00)	0 (0.00)	0 (0.00)	0 (0.00)
Control Group (n=20)	1 (5.00)	1 (5.00)	1 (5.00)	1 (5.00)	4 (20.00)
χ^2^					4.444
P					0.035

## Discussion

Congestive Heart Failure (CHF) is associated with myocardial injury caused by myocardial infarction, cardiomyopathy, and related inflammations. These injuries lead to changes in myocardial structure and function, leading to ventricular filling [8] [9]. Clinically, CHF primarily manifests as fluid retention and breathing difficulties. Patients with CHF usually have a history of heart disease, and targeted treatment can effectively improve patient outcomes and survival rates [10] [11]. Patients suffering from coronary heart disease may present with systolic CHF, and effective treatment can halt the disease’s progression [12]. Studies have shown that hypertension can trigger the development of diastolic CHF [13]; effectively controlling blood pressure can reduce the incidence of CHF. Clinical treatment of CHF goes beyond simply improving survival and reducing post-treatment complications. Enhancing a patient’s quality of life is also vitally important [14] [15]. Therefore, when choosing drugs for CHF treatment, considerations should be made based on both the patient’s physical conditions and the characteristics of the medication.

Past treatments for Congestive Heart Failure (CHF) in western medicine primarily utilized medications such as Sacubitril/Valsartan, Spironolactone, Furosemide, and Metoprolol Succinate. Sacubitril/Valsartan acts as an inhibitor of the angiotensin receptor, reducing blood pressure, alleviating cardiac load, and mitigating symptoms of heart failure [16]. As a commonly used diuretic in clinical practice, Spironolactone can suppress myocardial interstitial fibrosis and protect cardiac function [17]. Furose mide, commonly used for diuretic and edema-reducing purposes, can promote urine output to mitigate fluid and sodium retention in the body, thus eliminating symptoms such as chest tightness, shortness of breath, and bloating caused by heart failure [18]. It aids in expelling excess bodily water, reducing blood volume, dilating blood vessels, and inhibiting sympathetic nerve excitability. Metoprolol succinate is a beta-blocker that protects cardiomyocytes by alleviating intracardiac calcium overload, reducing myocardial damage and further improving cardiac function [19]. Although the aforementioned drugs can alleviate the clinical symptoms of CHF patients to some extent, the overall therapeutic effect is suboptimal, accompanied by certain adverse reactions, restricting their clinical application.

In recent years, Traditional Chinese Medicine (TCM) has demonstrated significant efficacy in treating Congestive Heart Failure (CHF). TCM classifies CHF under the categories of palpitations, edema, phlegmy drink, and asthma, with Qi deficiency and blood stasis being one of the most common symptom patterns [20]. TCM considers the pathological mechanism of CHF to be rooted in deficiency and excess, with kidney Yang deficiency as the root and subsequent stagnation of blood and phlegmy drink as the branches [21]. Research conducted by Ma et al. [22] observed that treating CHF patients with Chinese herbal medicine improved their therapeutic effectiveness and heart function. This study likewise found that CHF patients experienced better treatment outcomes and improved cardiac function indicators when treated with Chinese herbal medicine.

The traditional medicine applied in the present study is consisted of 15 g of Chaihu, 10 g of Xiangfu, 30 g of Astragalus, 15 g of Cornu Cervi Panto trichum, 10 g of Maidong, 10 g of seaweed, 10 g of Pu huang, 30 g of Lutong, 10g of peach kernel, 10 g of safflower, 10 g of Rhizoma Ligustici Chuanxiong, and 10 g of Bitter ginseng. Research conducted by Wen et al. [23] found that Chinese herbal medicine treatment could improve the quality of life and enhance exercise endurance in CHF patients. Especially, the protective role of the active ingredients in the Astragalus against heart failure has been widely verified [13]. This study similarly observed that Chinese herbal medicine treatment could reduce the MLHFQ score of CHF patients and extend their 6MWT results. The patient’s heart failure symptoms were significantly improved, reducing the impact of the disease on daily life, thereby improving the quality of life and exercise endurance.

Inflammatory factors such as hs-CRP can cause hypoxia and vascular spasms by activating inflammatory cells. TNF-α, a negative inotropic factor, can cause left ventricular dilation and enlarged end-diastolic diameter. IL-6, through coupling protein gpl30, can induce myocardial cell hypertrophy and independently regulate cardiac function [24]. NT-proBNP is a product generated when BNP is cleaved in the human body and is a peptide hormone secreted by ventricular myocardial cells that can reflect myocardial tension, and it holds significant diagnostic value for CHF. The results of this study demonstrate that traditional Chinese medicine treatment can reduce the levels of serum inflammatory markers and NT-proBNP in patients with CHF. The study results showed that Chinese herbal medicine could reduce serum inflammatory markers in CHF patients. This is because Astragalus can effectively enhance and regulate the immune function of the body, has a wide range of antibacterial and inflammation control effects, as well as regulating blood lipids, anti-aging, antioxidant, anti-radiation, etc; bitter ginseng in the bitter ginseng contained in the bitter ginseng alkaloids and other nutrients can improve the contractile function of the myocardium, enhance the ability of myocardial oxygenation, and have antibacterial and anti-inflammatory effect, may be the formula to inhibit the level of serum markers and NT-BNP reasons. Research conducted by Zeng et al. [25] found that Chinese herbal medicine could reduce the incidence of adverse reactions in CHF patients. This study also found that adverse reactions were fewer in CHF patients treated with Chinese medicine, as its main ingredients are natural plants, animals, and minerals with lower toxicity and milder pharmacological effects after processing, leading to a lower incidence of adverse reactions and high safety.

In summary, CHF patients who undergo treatment with Traditional Chinese Medicine demonstrate better therapeutic effectiveness relative to those receiving standard treatment. This approach further improves the patients’ heart function, enhances their exercise tolerance and quality of life, lowers serum marker and NT-BNP levels, and reduces the occurrence of adverse reactions. However, there are certain limitations to this study. Firstly, the small sample size (40 patients) limits the statistical power and generalizability of the findings. Larger-scale randomized controlled trials are necessary to validate these results. Furthermore, the short follow-up period (3 months) prevents the assessment of long-term efficacy and safety, including critical outcomes such as mortality and hospital readmission rates. Secondly, while this study focused on the therapeutic efficacy of TCM, the mechanisms underlying its effects were not deeply explored. Future studies incorporating molecular biology and omics technologies are recommended to elucidate the specific pathways by which TCM regulates inflammatory markers and improves cardiac function. The specific mechanism through which Traditional Chinese Medicine suppresses inflammatory responses in treating CHF patients requires more in-depth analysis in the future.

## Dodatak

### Funding

The present study was supported by the Major science and technology project of Changzhou Health Commission (No. ZD202124).

### Acknowledgement

Not applicable.

### Conflict of interest statement

All the authors declare that they have no conflict of interest in this work.
